# Novel PARP-1 Inhibitor Scaffolds Disclosed by a Dynamic Structure-Based Pharmacophore Approach

**DOI:** 10.1371/journal.pone.0170846

**Published:** 2017-01-25

**Authors:** Salete J. Baptista, Maria M. C. Silva, Elisabetta Moroni, Massimiliano Meli, Giorgio Colombo, Teresa C. P. Dinis, Jorge A. R. Salvador

**Affiliations:** 1 Laboratory of Pharmaceutical Chemistry, Faculty of Pharmacy, University of Coimbra, Coimbra, Portugal; 2 Center for Neuroscience and Cell Biology, University of Coimbra, Coimbra, Portugal; 3 Istituto di Chimica del Riconoscimento Molecolare, CNR, Milano, Italy; 4 Faculty of Pharmacy, University of Coimbra, Coimbra, Portugal; University of Parma, ITALY

## Abstract

PARP-1 inhibition has been studied over the last decades for the treatment of various diseases. Despite the fact that several molecules act as PARP-1 inhibitors, a reduced number of compounds are used in clinical practice. To identify new compounds with a discriminatory PARP-1 inhibitory function, explicit-solvent molecular dynamics simulations using different inhibitors bound to the PARP-1 catalytic domain were performed. The representative structures obtained were used to generate structure-based pharmacophores, taking into account the dynamic features of receptor-ligand interactions. Thereafter, a virtual screening of compound databases using the pharmacophore models obtained was performed and the hits retrieved were subjected to molecular docking-based scoring. The drug-like molecules featuring the best ranking were evaluated for their PARP-1 inhibitory activity and IC_50_ values were calculated for the top scoring docked compounds. Altogether, three new PARP-1 inhibitor chemotypes were identified.

## Introduction

Poly(ADP-ribose) polymerases (PARPs) comprise a group of enzymes that share the ability to catalyze the attachment of ADP-ribose moieties to specific acceptor proteins and transcription factors, using nicotine adenine dinucleotide (NAD^+^) as a substrate [1].

PARP-1 is the best characterized isoform among the PARP family members and is responsible for 85%-90% of poly(ADP-ribosylation) activity [2]. It plays an active role in several biological processes, including inflammation, hypoxic response, transcriptional regulation, maintenance of chromosome stability, DNA repair, and cell death [2–6]. The participation of PARP-1 in DNA repair granted it the designation of *guardian angel* of DNA [7]. This nuclear enzyme recognizes and binds to DNA strand-breaks via an N-terminal region, which promotes a conformational change in the C-terminal catalytic domain. As a result, this domain becomes activated, exposing the activation site to NAD^+^ and leading to the poly(ADP-ribosylation) of many targets, including histones and PARP-1 itself [3, 8].

The development of PARP-1 inhibitors as a therapy for several pathologies has been pursued, with special relevance in cancer and ischemic diseases [1]. The by-product of NAD^+^ cleavage, nicotinamide, has been used as the structural basis for the discovery of PARP-1 inhibitors. A large number of nicotinamide/benzamide derivatives have been studied, and some compounds have entered clinical trials as chemopotentiators in combination with anticancer drugs, as well as stand-alone agents in tumors with BRCA 1/2 mutations, taking advantage of synthetic lethality [8–11]. The drug candidate olaparib (Lynparza^TM^) was recently approved as the first PARP1/2 inhibitor to treat advanced ovarian cancer in women with defects in the *BRCA1/2* genes, who were previously treated with three or more chemotherapeutic lines [12]. Nevertheless, a polypharmacological profile has been assigned to PARP-1 drug candidates. The inhibition of other PARP isoforms, or even the interaction with other inter-family targets, was noted for several inhibitors in clinical trials [1, 13]. Moreover, olaparib was reported to act as a substrate of the p-glycoprotein efflux pump, one of the mechanisms that are associated with resistance to PARP inhibitors [8, 14]. Clearly, more in-depth studies of the determinants of the PARP-1 recognition features are needed to develop novel and more selective PARP-1 inhibitors.

Computational methods have emerged as an important tool in drug discovery, as they disclose key features in the ligand-receptor binding interactions and allow the screening of large compound libraries, thus saving time and resources [15]. Moreover, molecular dynamics (MD) simulations have become an important method to solve one of the biggest challenges in drug discovery, i.e., the use of a single crystal structure of a protein to predict the putative ligand-binding site, not considering the target plasticity that is involved in ligand binding [16]. Different studies have combined MD with pharmacophore modelling, taking advantage of receptor flexibility to build structured-based pharmacophore models. In general, a wide array of drug discovery examples based on this approach have shown that they provide a better prediction of truly active compounds compared with inactive ones and are able to find potential leads for different targets under investigation [17–22].

In this work, a dynamic structure-based pharmacophore methodology was pursued to identify new scaffolds with PARP-1 inhibitory activity. A virtual screening of the available compounds databases was performed using the pharmacophore models generated, and the top scoring compounds identified by molecular docking studies were validated through an *in vitro* PARP-1 inhibition assay.

## Materials and Methods

### MD simulations

Four inhibitors that bound to the PARP-1 catalytic domain were retrieved from the Protein Data Bank (PDB codes: 2RCW, 3GN7, 3GJW, 3L3L). Crystal structures were processed using the Protein Preparation Wizard tool in Maestro Suite (Release 2013-1-9.4, Schrödinger, LLC, New York, NY, 2013). Water molecules were removed and atom types were assigned.

For each ligand-bound system, MD simulations in explicit water were performed using the Amber package, v12. Amber FF99SB [23] and Generalized Amber Force Field (GAFF) [24] were assigned to the protein and ligands, respectively. Systems were solvated with TIP3P water molecules [25] in a truncated octahedral box, counter ions were added to neutralize the system net charge, and the periodic boundary conditions were applied. The final systems were composed of ~ 33400 atoms.

After minimizations, systems were submitted to an equilibration phase for 1 ns in NVT conditions, in which protein and ligand atoms were position restrained with a constant force of 10 kcal/mol, to allow relaxation of the solvent molecules. A final production phase of 20 ns was performed and trajectory snapshots were saved at every 10 ps, for each system. The Langevin temperature equilibration scheme was used to keep the temperature constant (300 K), and a constant pressure periodic boundary was applied (1 atm). Electrostatic and Lennard-Jones forces were assessed using the Particle Mesh Ewald summation method [26] and a cut-off of 10 Å, respectively. The SHAKE algorithm was applied to constrain bonds that involved hydrogen atoms.

GROMACS [27] was used to perform the trajectory analysis. For each system, a conformational cluster analysis was carried out using a cut-off of 0.06–0.07 nm RMSD (root mean square deviation) between the backbone superposition of different structures. All snapshots saved from each MD trajectory were extracted and used to perform cluster analysis. To characterize the dynamics features of active site-ligand interactions in the PARP-1 catalytic domain, only the residues that were set to 5 Å around the inhibitor were taken into account in the cluster analysis.

### Structure-based pharmacophore modelling and validation

Four different pharmacophore models were built based on the protein-ligand interactions observed after MD simulations. For each ligand-bound system, the clusters that represented more than 80% of the protein structural variability for each simulated system were selected to generate structure-based pharmacophores, using The Receptor-Ligand Pharmacophore Generation protocol of Accelrys Discovery Studio v3.5 (DS), Accelrys, San Diego, USA. This protocol uses receptor-ligand interactions to create selective pharmacophore models. Hydrogen bond acceptor (HBA), hydrogen bond donor (HBD), hydrophobic (HY), negative ionizable (NI), positive ionizable (PI), and ring aromatic (RA) features, as well as the excluded volume spheres set to 5 Å around the inhibitor, were considered in the generation of the pharmacophore models. The hypotheses created were validated by a set of known PARP-1 ligands and decoys obtained from Database Useful Decoys Enhanced (DUD_E)—http://dude.docking.org/, using the validation option incorporated in the protocol. For each cluster, the hypotheses were ranked based on specificity and sensitivity, and the one that presented the best accuracy was chosen.

The best hypotheses that were retained for each cluster of a specified complex were superimposed, and the average coordinate point for each feature, including the excluded volumes spheres, was determined.

Four final pharmacophore models were created, one for each complex. As a final validation, the pharmacophore models were screened against the PARP-1 actives and decoys, to evaluate how well they discriminate active molecules from inactive ones. Moreover, the presence of chemical features that were essential for the interaction with key residues in the PARP-1 catalytic domain was taken into account in the validation of the pharmacophore models.

### Database preparation and pharmacophore-based virtual screening

The National Cancer Institute (NCI)– https://cactus.nci.nih.gov/download/nci/ and DrugBank (http://www.drugbank.ca/) databases were downloaded. PARP-1 ligands and decoys were downloaded from the DUD_E database. Seven hundred and forty-two actives (affinity ≤1μM) and 30403 decoys (affinity ≥30 μM) were divided and converted into two databases, DUD_PARP1_ligands and DUD_PARP1_decoys, respectively. The “FAST” conformational analysis model of the catDB program was used to build the four databases, and a maximum of 255 conformations were generated for each molecule.

The four pharmacophore models obtained were used to screen the NCI and DrugBank databases using the “fast flexible database search” settings of Catalyst, to search for novel structural scaffolds with an ability to inhibit PARP-1.

The retrieved hits were subjected to different drug-like filters. Lipinski’s rule of five [28] and the modified Veber rule [29] (not more than 7 routable bonds) were applied. A maximum polar surface area was set to 140.

### Docking Studies

The docking studies were performed using Glide (version 5.8). Standard precision (SP) and extra precision (XP) modes were applied, using the OPLS-AA force field [30].

The protein retrieved from the crystal structure of A620223 binding to PARP-1 (PDB code: 2RCW) was used to define the binding site. The Preparation Wizard tool was applied and all water molecules were removed from the crystal. A 15×15×15 Å receptor grid centered on the co-crystalized ligand was generated.

The final selected hits, as well as a set of know PARP-1 inhibitors (downloaded from BindingDB database (http://www.bindingdb.org)), were prepared using the LigPrep module (Schrödinger, LLC, New York, NY, 2013). The pH was set to 7.4 and a maximum of 5 stereoisomers per ligand were generated. The lowest energy ring conformation was kept for each stereoisomer.

An initial docking was performed using the SP-mode and 25 poses were kept for each molecule. A cut-off based on the docking score of reference PARP-1 inhibitors was used, and ligands with the highest score were subjected to XP docking.

### PARP-1 enzyme assay

PARP-1 inhibition was evaluated using the HT Universal Colorimetric PARP Assay kit (Catalog #4677-096-K; Trevigen, Gaithersburg, MD, USA), in line with the instructions provided by the manufacturer. The assay evaluates the incorporation of biotinylated poly(ADP-ribose) onto histones proteins in a 96-well plate. Briefly, 10 μL of the test compounds were mixed with 15 μL of PARP-1 enzyme (0.5 U) into rehydrated histone-coated wells for 10 min at room temperature. Subsequently, 25 μL of PARP cocktail containing biotinylated NAD, activated DNA, and PARP buffer were added, and the solutions were incubated again for 60 min. After washing the wells, the detection reaction was performed according to the manufacturer’s protocol and absorbance was recorded at 450nm in a synergy HT plate reader. Stock solutions of the test compounds were prepared in dimethyl sulfoxide (DMSO) and serially diluted to the required concentrations with 1× PARP buffer. To assess the effect of the vehicle on enzyme activity, parallel experiments were performed by substituting the test compound with an equivalent volume of DMSO. IC_50_ values for the most promising hits were determined by plotting the inhibition data of each compound at different concentrations against the log of the concentration of the inhibitor, using the GraphPad Prism software, version 5. At least six different concentrations of the test compounds were used. A minimum of three independent assays were performed for each sample, and the results are displayed as mean ± standard error of the mean (SEM).

### Nuclear Magnetic Resonance (NMR) studies

1D and 2D NMR structure elucidation of the NSC86342, NSC121848, and NSC131753 compounds was obtained using a Brucker Digital NMR-Avance 400 spectrometer, with CD_3_OD as the internal standard.

### NSC131753 MD simulations

MD simulations were performed using (*R*)-NSC131753 and (*S*)-NSC131753 complexed with the PARP-1 catalytic domain, using top XP Glide poses as input structures. The MD simulations were performed using the protocol described above for the four complexes taken from PDB, with equilibration and production phases of 50 ps and 100 ns, respectively. Three replicas (100 ns) were run for each system with different initial velocities, to increase sampling.

MD trajectory analysis was performed using the GROMACS package.

## Results and Discussion

### Structural and dynamic characterization of different complexes with known inhibitors

**Fig 1:**
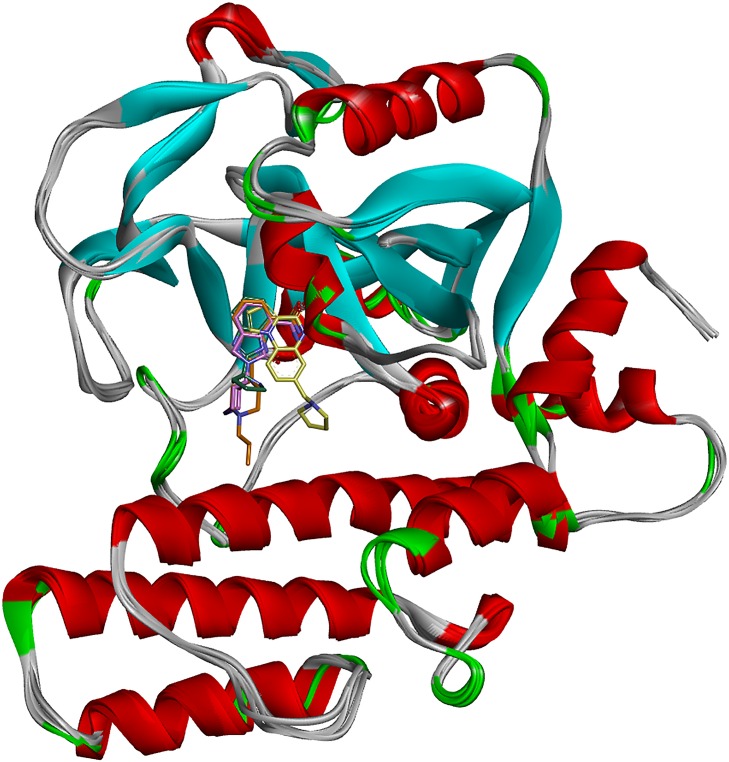
Superposition of the starting crystal structures of 2RCW, 3L3L, 3GN7 and 3GJW complexes.

In this work, MD simulations with different known small-molecule inhibitors were carried out to characterize the dynamic features of active site-ligand interactions in the PARP-1 catalytic domain. In this context, the aim of MD simulations was not the full sampling of the events underlying complex formation or the exploration of ligand induced conformational changes, which can be considered as being absent, given the high global similarity of the starting crystal structures, with a maximum RMSD (as calculated on protein backbone atoms) of 0.62 Å (**Fig 1**). Rather, a comparative analysis of the trajectories from the different complexes was used to identify the salient features of the dynamic adaptation of PARP-1 to diverse active site inhibitors. Our general goal was to characterize the cross-talk between the ligands and the protein and highlight the binding interactions that were consistently preserved in multiple configurations, in addition to the ones that were immediately evident from crystal structures. Those conserved binding interactions were then used to develop dynamic pharmacophore models aimed at expanding the chemical diversity space of PARP-1 inhibitors.

**Fig 2:**
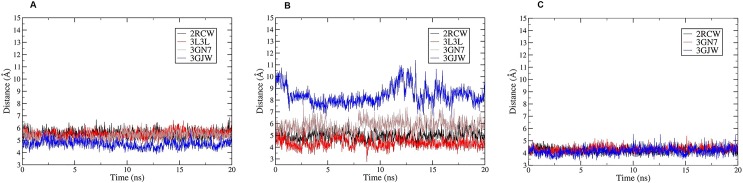
The plot distances involving the tyrosine residues and ligands along MD trajectories. A) Distance between Tyr246 centroid π ring and the aromatic core of ligands. B) Distance between Tyr228 centroid π ring and the protonate amine moiety of each ligand. C) Distance between the centroid of amide group (formed by the CO of Tyr235 and N of Phe236) and phenyl ring of each ligand.

**Fig 3:**
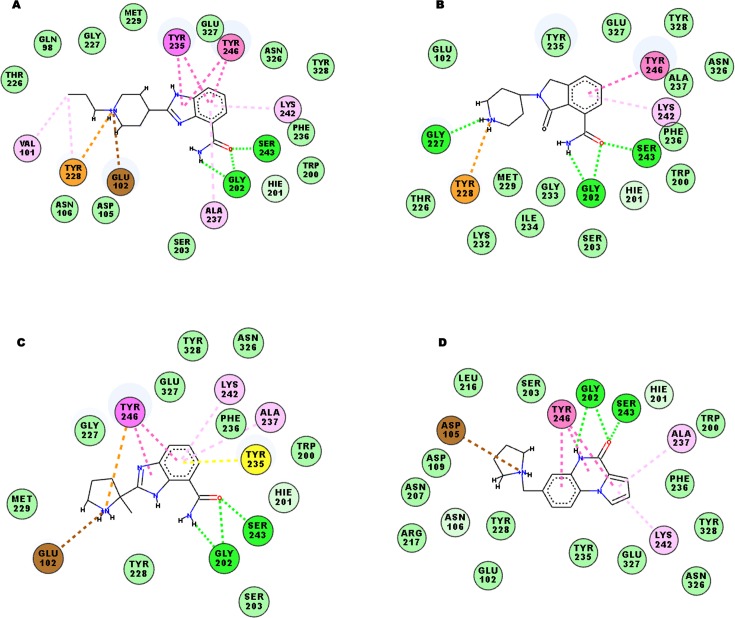
2D-Ligand interaction diagrams for each ligand complexed with PARP-1 catalytic domain along MD run. Dashed lines represent interactions between binding site residues and bounded ligands. Green color pointed to hydrogen bond interactions; Orange indicates π-cation interactions; pink denotes π-π stacked; yellow pointed to amide-π interactions; light pink denotes hydrophobic interactions (alkyl and π-alkyl); brown indicates charge-charge interactions, and turquoise residues indicate van der waals interactions. A) 2RCW. B) 3L3L. C) 3GN7). D) 3GJW.

The analysis of the main clusters revealed that the key interactions that were present in the co-crystal structures of PARP-1 with four different inhibitors were conserved. Such interactions consisted of three stable hydrogen bonds: two between the amide backbone of Gly202 and the amide moiety of the inhibitors and one between the OH group of Ser243 and the carbonyl group of inhibitors, as presented in **Table 1A**, as well as a π-π stacking interaction involving Tyr246 and the aromatic core of the ligands. Furthermore, MD simulations showed that the tyrosine residues present in the binding site were involved in different π-interactions. To define the importance of the tyrosine residues, the contacts between Tyr228, Tyr235, and Tyr246 and the ligands were monitored during 20 ns MD run (**Fig 2**). Depending on the bound inhibitors, different tyrosine residues were engaged. Tyr228 appeared to be essential for the π-cation interaction with the protonated amine moiety of the 2RCW and 3L3L ligands, with occupancy of 99% and 100%, respectively, during MD trajectories. For the 3GN7 inhibitor, both Tyr228 and Tyr246 were implicated in this type of interactions, with occupancy of 65.45% and 47.5%, respectively. Moreover, Tyr235 and Phe236 were involved in amide-π interactions with the phenyl rings of the 2RCW, 3GN7, and 3GJW ligands. In addition, Ala237 and the alkyl side chain of Lys242 participated in hydrophobic interactions with the phenyl ring of each inhibitor along the MD trajectory for all ligand-bound systems analyzed (**Table 1B**). The protonated amine group of each ligand also appeared to be important for the establishment of charge-charge interactions with some charged residues present in the binding site, such as Glu102, Asp105, and Asp109 (**Table 1C**). This type of interaction was especially relevant for 3GJW. The 3GJW ligand was involved in charge-charge interactions with Asp105 (:OD1) for 84.65% of the MD run time. The main interactions for each complex along MD trajectories (the first cluster) are shown in **Fig 3.**

**Table 1:** Hydrogen bonds (A), hydrophobic interactions, namely alkyl and π-alkyl interactions (B), and charge-charge interactions (C) with greater occupancy during MD trajectories for 2RCW, 3L3L, 3GN7 and 3GJW complexes.

**A**			
**Hydrogen bonds**			
**PDB ID**	**Donnor**	**Acceptor**	**%Occupancy**
**2RCW**	Gly202:N	AAI:O1	40.05
	Ser243:OG	AAI:O1	64.80
	AAI:N3	Gly202:O	73.55
**3L3L**	Gly202:N	L3L:O2	54.75
	Ser243:OG	L3L:N3	38.85
	L3L:N3	Gly202:O	64.35
	L3L:N1	Gly227:O	23.80
**3GN7**	Gly202:N	3GN:O12	38.80
	Ser243:OG	3GN:O12	44.50
	3GN:N1	Gly202:O	61.70
**3GJW**	Gly202:N	GJW:O1	27.15
	Ser243:OG	GJW:O1	62.65
	GJW:N3	Gly202:O	74.80
**B**			
**Alkyl and π-alkyl interactions**			
**PDB ID**	**Residue**	**%Occupancy**
**2RCW**	Ala237	68.00
	Lys242	97.70
**3L3L**	Ala237	48.00
	Lys242	84.70
**3GN7**	Ala237	52.20
	Lys242	75.70
**3GJW**	Ala237	66.65
	Lys242	64.55
**C**		
**Charge-charge Interactions**			
**PDB ID**	**Residue**		**%Occupancy**
**2RCW**	Glu102	OE1	29.40
		OE2	32.50
	Asp105	OD1	30.30
		OD2	26.35
**3L3L**	Glu102	OE1	33.25
		OE2	37.60
**3GN7**	Glu102	OE1	29.15
		OE2	32.50
**3GJW**	Asp105	OD1	84.65
		OD2	19.65
	Asp109	OD1	30.95
		OD2	23.05

By exploring the diversity and the motion of the ligands, as well as the flexibility of the binding site residues, four structure/dynamics-based pharmacophores were generated based on the ligand-protein interactions that were monitored during the MD trajectories. The conserved hydrogen bonds, as well as the π-π stacking, the π-cation and charge-charge interactions observed to a higher extent during MD, were considered to generate structure-based pharmacophores.

For each complex, seven representative structures (matching more than 80% of the structural variability) were taken into account to build the pharmacophore models.

For all pharmacophore hypotheses, the Receptor-Ligand Pharmacophore Generation protocol in DS pointed key interactions between the PARP-1 catalytic domain and the ligand, and generated excluded volume spheres that were correlated with steric regions in the binding site that may not be engaged by the ligand substituent groups. In this context, the characterization of the mechanisms of the formation/disappearance of pockets around the ligands due to the immediate conformational response of the protein to known inhibitors can aptly indicate the positions at which the addition/modification of specific substituent groups may allow optimal extensions of binding interactions into previously uncharacterized regions.

**Fig 4:**
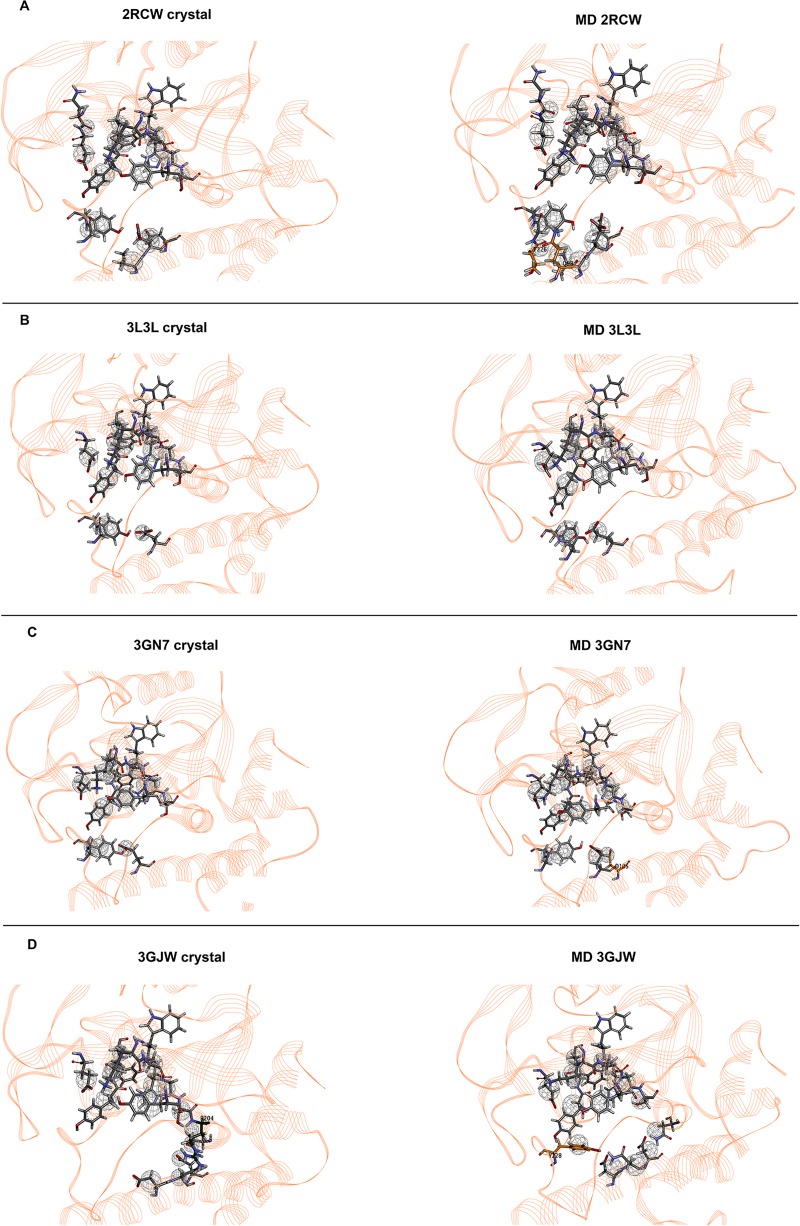
Representation of the PARP-1 binding site with excluded volume spheres. The excluded volume spheres, set to 5 Å around each inhibitor complexed with the binding site, were obtained from the crystal and the representative structure after MD simulations for 2RCW, 3L3L, 3GN7 and 3GJW complexes.

**Fig 5:**
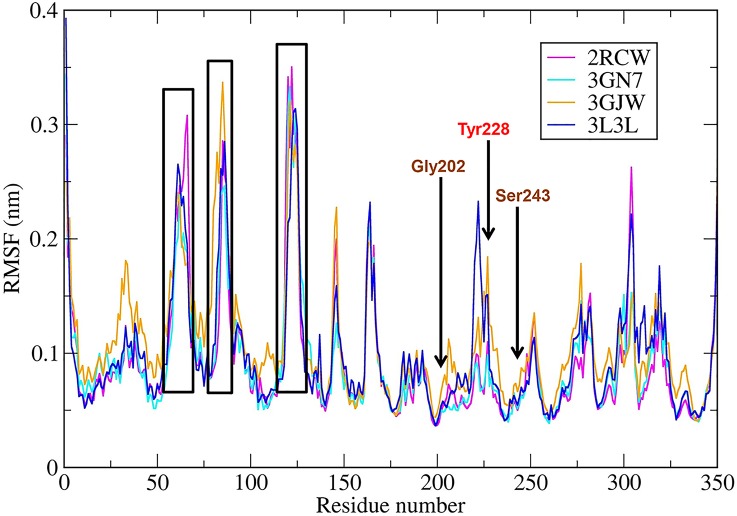
RMSFs of complexes along 20 ns MD run.

The comparison of the excluded volumes spheres obtained based on the crystal and the representative structure after MD simulations for each ligand-bound system (**Fig 4**) revealed that the excluded volume spheres were generally pointed to the same residues, especially in the nicotinamide binding pocket, which comprises residues such as Gly202, Ser243 and Tyr246 (**S1 Fig**). Analyses of root mean square fluctuation (RMSF) for all four complexes (**Fig 5**) revealed that the regions that contained nicotinamide binding residues were quite stable. The highest fluctuations were observed in loop regions (60–67; 78–94; and 118–128).

### Pharmacophore model building

**Fig 6:**
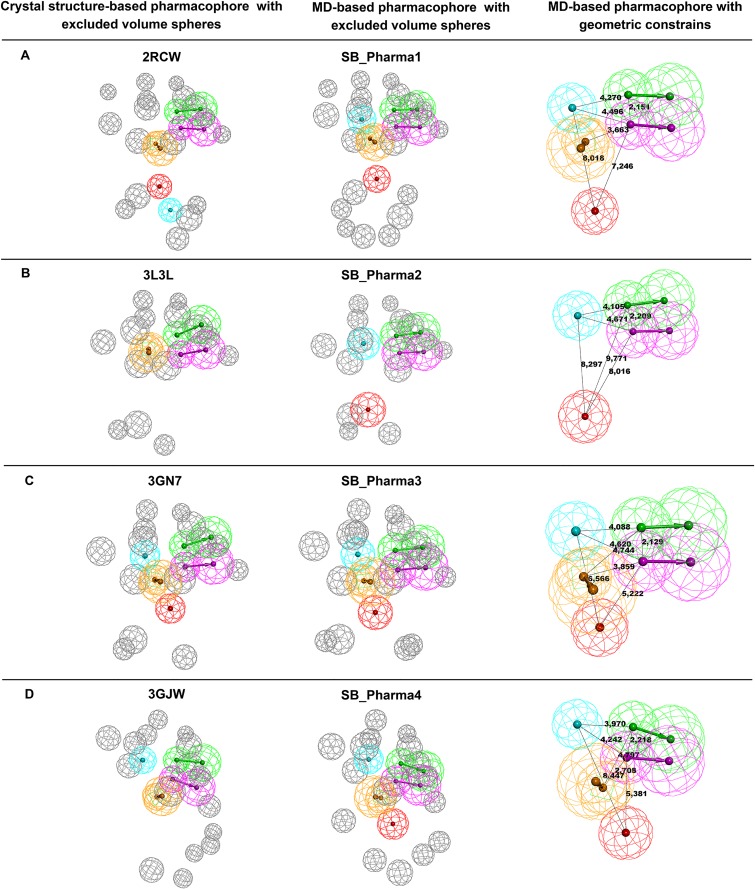
Representation of structure-based pharmacophore models obtained from crystal structures and the dominant conformations after MD simulations. Green color indicates hydrogen bond acceptor (HBA); magenta denotes to hydrogen bond donor (HBD); cyan shows hydrophobic center (HY); yellow indicates ring aromatic (RA); and red denotes to positive ionizable center (PI).

**SB_Pharma1**, which was based on the 2RCW complex, displayed five functional features, including an HBD and an HBA pointed to Gly202, an HY pointed towards Ala237 and Lys242 (alkyl side chain), an RA directed to Tyr246, and a PI pointed to Tyr228. Nineteen excluded volume spheres were identified, which represented an additional two spheres compared with the crystal structure. One of them was directed to Gln98 (side chain) and the other to Thr226 (backbone). **SB_Pharma2**, which was the pharmacophore model obtained from 3L3L, exhibited four features and 14 excluded volume spheres, pointed to the same residues of the crystal structure. One HBA and one HBD directed to Gly202, an HY pointed towards Ala237 and Lys242 (alkyl side chain), and a PI pointed to Tyr228 were observed. **SB_Pharma3** and **SB_Pharma4** were generated from 3GN7 and 3GJW, respectively. Both pharmacophore models exhibited three similar features: an HBA and an HBD pointed to Gly202, an HY center directed towards Ala237 and Lys242 (alkyl side chain) and an RA also directed to Tyr246. A PI center was pointed to Tyr228 or even Tyr 246 in **SB_Pharma3**, and to Asp105 in **SB_Pharma4**. Excluded volume spheres (15 and 16, respectively) were also identified. **SB_Pharma3** displayed an extra excluded volume (compared with the 3GN7 crystal structure) pointing towards the Asp105 side chain that changed side chain orientation during the MD simulation (**S2 Fig**). Moreover, **SB_Pharma4** also showed an additional excluded volume sphere, directed to Tyr228, which side chain exhibited considerable flexibility along the MD trajectory (**Figs 4D and 5**). The excluded volume directed to Arg204, which was observed in the 3GJW crystal structure, was not set in the final pharmacophore model obtained after MD. As illustrated in **Fig 3D**, pointing to this active site residue did not appear to be essential for productive interaction. The final structure-based pharmacophore models (**SB_Pharma1**, **SB_Pharma2**, **SB_Pharma3**, and **SB_Pharma4**) obtained from the superposition of dominant conformations for each ligand-bound system, as well as those obtained from PDB crystal structures, are elucidated in **Fig 6**.

The analysis of four structure-based pharmacophore models supported the essential role of Gly202 as an HBD and HBA, as well as the presence of important hydrophobic residues, namely Ala237 and Lys242 (alkyl side chain). Moreover, Tyr228, Tyr235, and Tyr246 were shown to be important for the establishment of different types of π-interactions. The aromatic feature directed to Tyr246, for instance, was necessary to mimic the relevant role of stacking interactions in driving effective binding to the PARP-1 catalytic domain. Finally, the presence of charge-charge interactions mediated by charged residues, as exemplified by Asp105, may be important for the identification of additional interactions that increase the binding affinity between the ligand and the protein.

### Pharmacophore-based virtual screening and validation

The four pharmacophore models were validated against DUD_PARP1_ligands and DUD_PARP1_decoys, which were generated by Catalyst, and both sensitivity and specificity were calculated (**Table 2**). Sensitivity was related to the fraction of PARP-1 binders that correctly fit the pharmacophore models. Specificity was related to the fraction of molecules that did not fully fit the pharmacophore hypotheses and were identified as decoys. The comparison of the values obtained for the four structure-based pharmacophore models showed that **SB_Pharma1** and **SB_Pharma4** displayed a better accuracy compared with the already good one characterizing all pharmacophore models, in general. On such bases, all four hypotheses generated were used to screen the NCI and DrugBank databases. However, to increase the ability to distinguish between active and inactive molecules, only hits with fit values above 2.0 and those that were retrieved by more than one pharmacophore model (in which at least one of them displayed the best accuracy (**SB_Pharma1** or **SB_Pharma4**)), were retained for further docking studies. Overall, 915 and 175 hits were obtained from the NCI and DrugBank databases, respectively.

**Table 2:** Statistical data of structure-based pharmacophore models.

**Pharmacophore Model**	**TA**	**TI**	**TP**	**TN**	**FP**	**FN**	**Se**	**Sp**	**Acc**
**SB_Pharma1**	742	30403	346	27926	2477	396	0.466	0.918	**0.908**
**SB_Pharma2**	742	30403	364	26037	4366	378	0.491	0.856	0.848
**SB_Pharma3**	742	30403	275	25873	4530	467	0.371	0.851	0.840
**SB_Pharma4**	742	30403	225	28073	2330	517	0.303	0.923	**0.908**

TA: Total number of actives; TI: Total number of inactives; TP: True positives; TN: True negatives; FP: False positives; FN: False negatives; Se: Sensitivity; Sp: Specificity; Acc: Accuracy.

Importantly, inspection of the retrieved hits identified 3 known PARP-1 inhibitors among the 175 molecules that were obtained from the screening of the DrugBank database: DB0372 (FR257517) [31], DB07787 (FR255595) [32], and D08348 (PJ34) [1]. It is worth noting here that these ligands were not part of the initial training set of ligands that was used to start MD simulations and pharmacophore design. The presence of these inhibitors, which have a higher potency regarding the inhibition of PARP-1 activity, constituted a first important validation of the capacity of our pharmacophore models to recapitulate the chemical and stereoelectronic determinants that underlie the activity of drug molecules.

### Docking studies

**Fig 7:**
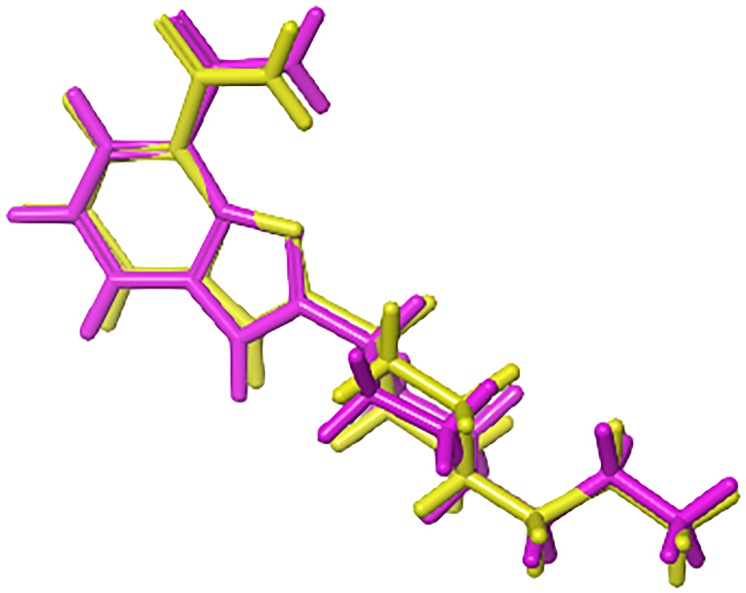
Superposition of the docked pose (magenta) of A620223 with its crystal structure conformation (yellow).

The overall 1090 retrieved hits (from the NCI and DrugBank databases) were docked at the PARP-1 binding site using Glide SP-mode. To validate and optimize the docking parameters, A620223 co-crystalized with the PARP-1 catalytic domain (PDB code: 2RCW), as well as 14 reference PARP-1 inhibitors, were re-docked. The SP docking results showed that the binding pose of A620223 in the crystal could be optimally reproduced, with an RMSD of 0.64 Å (**Fig 7**). Furthermore, the top poses of hits retrieved from the NCI and DrugBank databases were inspected, and a docking score cut-off of -7 was applied, based on the docking score range of the PARP-1 inhibitors that were docked ([-7.5;-11.6]). The remaining compounds were subjected to a second docking run, using Glide XP-mode. To select promising hits, a visual inspection of the compounds was performed. The interaction with key residues, such as Gly202 and Tyr246, as well as the structural diversity between the molecules, was taken into account when choosing potential hits. A total of 60 compounds were chosen for further evaluation.

### PARP-1 inhibition and structure-activity relationship

**Fig 8:**
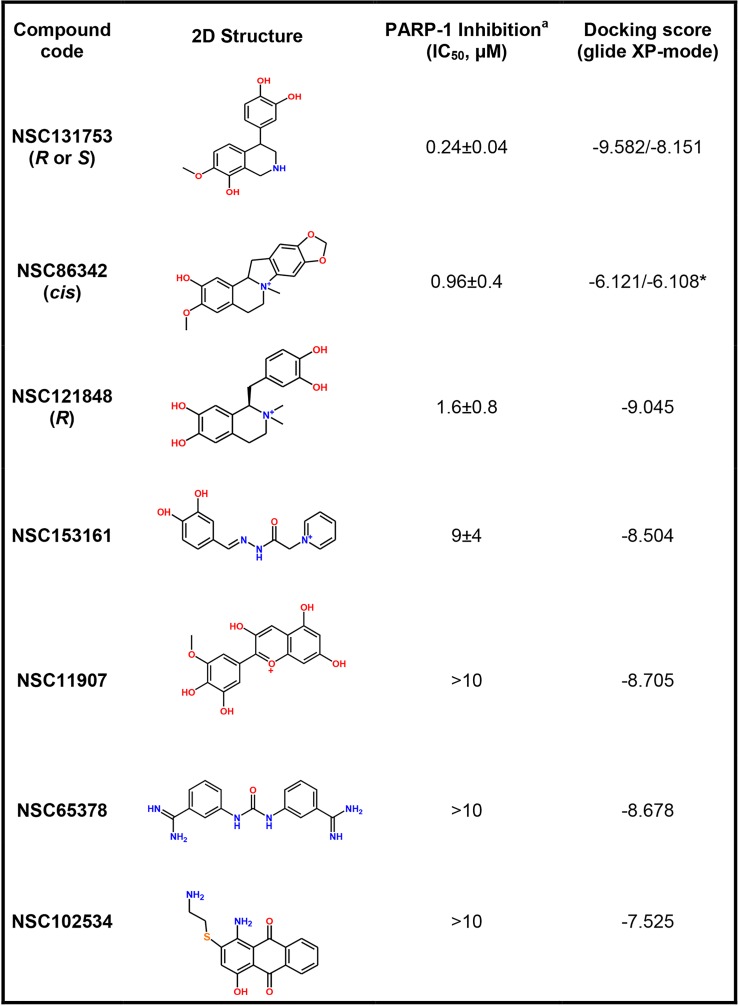
PARP-1 inhibitory activity and docking score data for the most promising hits. ^**a**^) PARP-1 inhibition was determined using HT Universal Colorimetric PARP Assay Kit (Cat #4677-096-k). *Docking score values of both possible **NSC86342** *cis* diastereomers.

**Fig 9:**
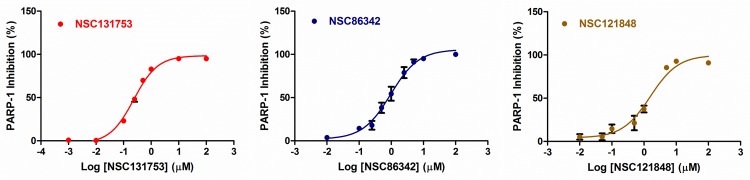
Dose-response curves of the three most promising hits. Each data point represents the mean ± SEM of at the least three independent experiments.

The HT Universal Colorimetric PARP Assay Kit was used to screen and to determine the IC_50_ values of the promising hits obtained. Only 39 compounds among the 60 chosen above were effectively tested, because of commercial availability or solubility problems. After an initial screening at a concentration of 100 μM, seven compounds displayed a PARP-1 inhibition activity >90%. A new screening at 10 μM was performed. The IC50 was determined for the most promising hits (**Fig 8**and **Fig 9**).

**Fig 10:**
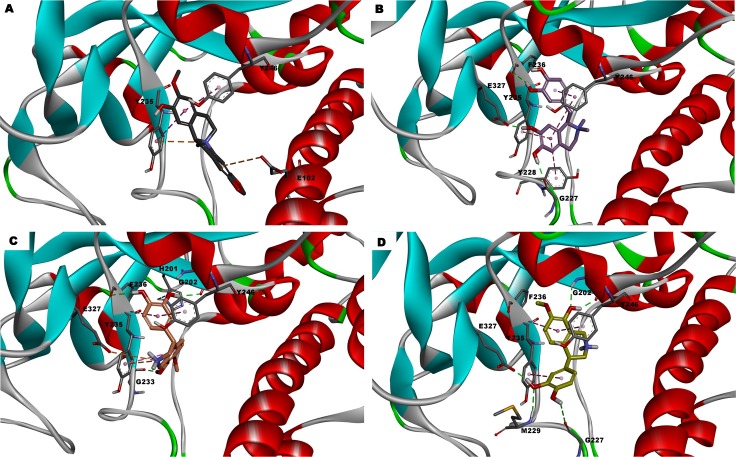
The binding mode of the most promising PARP-1 inhibitors at the PARP-1 catalytic domain. The molecular interactions of the top scored poses were displayed. A) NSC86342. B) NSC121848. C) (*R*)-NSC131753. D) (*S*)-NSC131753.

Among the promising molecules, three of them exhibited one or more chiral centers. To determine which isomer was acquired from NCI, the NSC86342, NSC131753, and NSC121848 compounds were characterized by NMR. The ^1^H NMR and ^13^C NMR spectra showed that only an isomeric form was present for each sample (**S1 File**). Moreover, the NOESY spectrum allowed the characterization of the enantiomeric form obtained for NSC121848. The H9 proton observed in the NOESY spectrum was correlated with both methyl groups at N1, which indicates that the (*R*)-enantiomer was present (**S2 File**). Similarly, in the NOESY spectrum of NSC86342, a correlation between H10 and the methyl group at N1was observed. This demonstrates that these two groups have the same orientation, which reveals that NSC86342 is a *cis* diastereomer (**S3 File**). As can be observed, there was a huge structural variability between the most promising compounds, with NSC131753 showing the highest PARP-1 inhibitory activity (IC_50_ = 0.24 μM). Moreover, PARP-1 inhibition was well correlated with the XP docking scores of three among the top four most promising hits (**Fig 8**). Despite the fact that NSC86342 showed the lowest docking score, it displayed π-cation and π-π interactions with key tyrosine residues (Tyr235 and Tyr246), which have been described as being essential for the binding of PARP-1 inhibitors to the catalytic domain. Moreover, some poses revealed π-charge interactions with Glu102, Glu327, and Tyr228. Taken together, these findings may explain the stability of this compound at the binding site and its high PARP-1 inhibitory activity (**Fig 8**and **Fig 10A)**.

As expected, all promising hits were involved in interactions with conserved binding residues, such as Gly202, Tyr246, or even Tyr235. The presence of donor-acceptor aromatic systems appeared to be essential for PARP-1 inhibition, which is in line with the hydrophilic environment that surrounded the NAD^+^ binding pocket, with a remarkable presence of aromatic residues (**S1 Fig**). Consequently, it is easy to understand why the best PARP-1 inhibition activities were displayed by molecules with an aromatic polycyclic skeleton with several HBAs or HBDs, such as NSC131753 and NSC121848 **(Fig 10B)**. The latter established hydrogen bonds with a vast number of residues, such as Phe236, Gly227, and Glu327, in addition to the π-cation and π-π interactions with the key Tyr235 and Tyr246 residues. Although the NMR analysis did not determine which NSC131753 enantiomer was evaluated, both (*R*) and (*S*) enantiomeric forms may be involved in different types of interactions with the catalytic domain, in spite of the differences in docking scores. Hydrogen bond interactions involving Gly233 and Phe236 were stablished with the (*R*)-enantiomer (**Fig 10C**), while Gly227 and Met229 were implicated in this type of interactions with the (*S*)-enantiomer (**Fig 10D**). Moreover, Glu327 played an important role in the interaction profile of both enantiomeric forms, by establishing π-cation interactions with (*R*)-NSC131753 and H-bond with (*S*)-NSC131753. Further insights into the binding mode of the two enantiomeric forms will be discussed in the description of the NSC131753 MD simulations analysis.

It is worth mentioning that anthraquinone derivatives, of which NSC102534 is an example, have been recently reported as being PARP-1 inhibitors [33]. The polycyclic aromatic core of these compounds was crucial for the interaction with the binding site (**S3 Fig**).

In addition, it is important to note that the hits with the most promising PARP-1 inhibitory activity, **NSC131753**, **NSC86342**, and **NSC121848** consist, to the best of our knowledge, in new PARP-1 inhibitor skeletons. These compounds shared the ability to interact not only with conserved nicotinamide-binding pocket residues, such as Gly202, Tyr235, and Tyr246, but also with some residues located on a donor-site loop (Gly215-Gly233), such as Gly227 (NSC121848), Tyr228 (NSC86342), and Met229 ((*S*)-NSC131753); this, could explain the binding mode stability and the relevant PARP-1 inhibition values observed.

**Fig 11:**
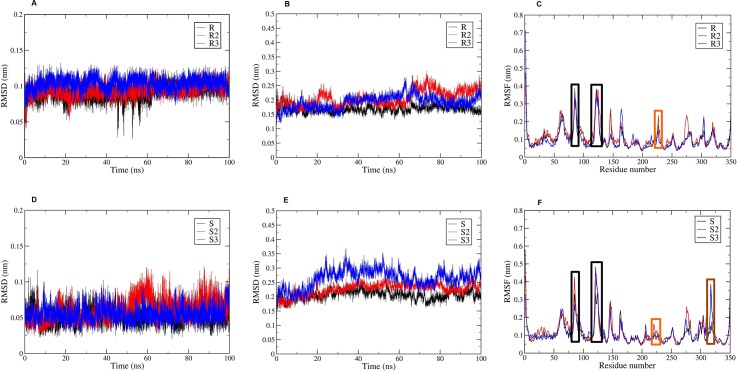
Conformational statistics obtained for (*R*)- and (*S*)-NSC131753, along 100 ns MD run. (A, D) RMSDs of ligands. (B, E) RMSDs of complexes. (C, F) RMSFs of Cα atoms of catalytic domain.

The most promising hit, **NSC131753**, contains a chiral center. Despite the performance of 1D and 2D NMR studies, it was not possible to identify the enantiomeric form evaluated against the PARP Assay kit. To determine which enantiomer is more stable at the binding site and to attest the interaction profile obtained from the docking studies, 100 ns long MD simulations were carried out for (*R*)- and (*S*)-NSC131753 complexed with the catalytic domain. The RMSD was lower for (*S*)-NSC131753 (around 0.05 nm compared with 0.09 nm for (*R*)-NSC131753), even though the two enantiomers revealed being quite stable (**Fig 11A and 11D**). However, the average RMSD calculated on the Cα atoms was lower and more stable for the (*R*)-enantiomer complex, along the three 100 ns MD replicas (**Fig 11B and 11E**)). Conversely, a similar RMSF distribution (**Fig 11C and 11F**) was observed for both enantiomeric forms, with the highest fluctuation observed in two loop regions of the catalytic domain (78–94; 118–128). Nevertheless, a highlighted mobility was observed from residues 317 to 322 in the RMSF plot of (*S*)-NSC131753, which was not observed for (*R*)-NSC131753. This may indicate a conformational change induced by the (*S*)-enantiomer. It is also worth noting that the D-loop residues (215–233) presented higher flexibility in the case of (*R*)-NSC131753 (at least 0.02 nm) compared with (*S*)-NSC131753 (around 0.015 nm). This difference may be due to the interaction of (*S*)-NSC131753 with Tyr228 (through π-π and π-cation interactions) and Met229 (hydrogen bond). In fact, the analysis of the interaction profile during MD showed that both enantiomers established an important number of interactions. Moreover, the main interactions proposed by the docking studies were maintained for both enantiomers, with high occupancy. Tyr235 and Tyr246 were involved in π-π interactions with both enantiomers, with occupancies above 65%. Glu327 was essential for the establishment of hydrogen bonding with both enantiomeric forms, and for charge-charge interactions with (*R*)-NSC131753, as demonstrated by the docking studies described above. MD trajectories analysis also revealed that Glu102 established a hydrogen bond interaction with the (*R*)-enantiomer for 41.5% of the MD run time, and with the (*S*)-enantiomer for 35.01%. A similar type of interaction was identified between ASP105 and (*S*)-NSC131753, with an occupancy of 38.55% along 100 ns MD simulations.

In summary, the data showed that both enantiomers were able to interact with the catalytic domain with relative stability, via different types of interactions with binding site residues, some of which were revealed only during MD simulations. An example of this is the interaction between (*S*)-NSC131753 and Tyr228, which was important to stabilize the D-loop and may explain the differences in docking scores observed between (*R*)- and (*S*)-NSC131753.

## Conclusions

A dynamic structure-based pharmacophore strategy was used to identify novel PARP-1 inhibitors. The pharmacophore models based on the interactions between the PARP-1 catalytic domain and four different inhibitors during MD simulations provided new insights in the ligand binding mode, taking into account the flexibility of both the enzyme and the ligand. Subsequently, the validated pharmacophore models were screened against two virtual compound libraries, to retrieve hits with novel chemical scaffolds. After molecular docking studies using Glide, the top scored drug-like molecules were tested against the PARP kit assay to determine PARP-1 inhibitory activity. Structurally diverse hits with important PARP-1 inhibitory activity were found. Moreover, the dynamic structure-based pharmacophore approach applied here led to the identification of three new PARP-1 inhibitor candidates with skeletons that had not been reported previously: **NSC86342**, **NSC131753**, and **NSC121848**. These candidates will be useful for guiding the further development of novel and more potent PARP-1 inhibitors.

## Supporting Information

S1 Fig Binding pocket residues of 2RCW. Nicotinamide (A) and adenine-ribose (B) binding residues are displayed in red and blue, respectively. Violet was used to show D-loop residues (C).
(PDF)

S2 Fig Asp105 side chain orientation in the crystal structure and after MD simulations (representative structure). (PDF)

S1 File ^1^H-NMR and ^13^C-NMR data for NSC86342, NSC121848 and NSC131753. (PDF)

S2 File NOESY spectrum of NSC121848. (PDF)

S3 File NOESY spectrum of NSC86342. (PDF)

S3 Fig **The binding mode of NSC153161 (A), NSC102534 (B), NSC65378 (C) and NSC11907 (D) at the PARP-1 catalytic domain.** The molecular interactions of the top scored poses were displayed.
(PDF)

## References

[1] Passeri D, Camaioni E, Liscio P, Sabbatini P, Ferri M, Carotti A, et al Concepts and Molecular Aspects in the Polypharmacology of PARP-1 Inhibitors. ChemMedChem. 2016;11(12):1219–26. 10.1002/cmdc.201500391 26424664

[2] Xu S, Bai P, Little PJ, Liu P. Poly(ADP-ribose) polymerase 1 (PARP1) in atherosclerosis: from molecular mechanisms to therapeutic implications. Med Res Rev. 2014;34(3):644–75. 10.1002/med.21300 24002940

[3] Rodriguez MI, Majuelos-Melguizo J, Marti Martin-Consuegra JM, Ruiz de Almodovar M, Lopez-Rivas A, Javier Oliver F. Deciphering the insights of poly(ADP-ribosylation) in tumor progression. Med Res Rev. 2015;35(4):678–97. 10.1002/med.21339 25604534

[4] Morales J, Li L, Fattah FJ, Dong Y, Bey EA, Patel M, et al Review of poly (ADP-ribose) polymerase (PARP) mechanisms of action and rationale for targeting in cancer and other diseases. Critical reviews in eukaryotic gene expression. 2014;24(1):15–28. Epub 2014/03/04. PubMed PubMed Central PMCID: PMCPmc4806654. 24579667 10.1615/critreveukaryotgeneexpr.2013006875 PMC4806654

[5] Scott CL, Swisher EM, Kaufmann SH. Poly (ADP-ribose) polymerase inhibitors: recent advances and future development. Journal of clinical oncology: official journal of the American Society of Clinical Oncology. 2015;33(12):1397–406. Epub 2015/03/18. PubMed Central PMCID: PMCPmc4517072. 10.1200/JCO.2014.58.8848 PMC4517072 25779564

[6] Verdone L, La Fortezza M, Ciccarone F, Caiafa P, Zampieri M, Caserta M. Poly(ADP-Ribosyl)ation Affects Histone Acetylation and Transcription. PLoS One. 2015;10(12):e0144287 Epub 2015/12/05. PubMed Central PMCID: PMCPmc4670112. 10.1371/journal.pone.0144287 26636673 PMC4670112

[7] Ferraris DV. Evolution of poly(ADP-ribose) polymerase-1 (PARP-1) inhibitors. From concept to clinic. J Med Chem. 2010;53(12):4561–84. Epub 2010/04/07. 10.1021/jm100012m 20364863

[8] Lupo B, Trusolino L. Review: Inhibition of poly(ADP-ribosyl)ation in cancer: Old and new paradigms revisited. Biochim Biophys Acta. 2014;1846(1):201–15. Epub 2014/07/16. 10.1016/j.bbcan.2014.07.004 25026313

[9] Curtin NJ, Szabo C. Therapeutic applications of PARP inhibitors: anticancer therapy and beyond. Molecular aspects of medicine. 2013;34(6):1217–56. Epub 2013/02/02. PubMed Central PMCID: PMCPmc3657315. 10.1016/j.mam.2013.01.006 23370117 PMC3657315

[10] Tangutoori S, Baldwin P, Sridhar S. Review: PARP inhibitors: A new era of targeted therapy. Maturitas. 2015;81(1):5–9. Epub2015/02/25. 10.1016/j.maturitas.2015.01.015 25708226

[11] Sistigu A, Manic G, Obrist F, Vitale I. Trial watch—inhibiting PARP enzymes for anticancer therapy. Molecular & cellular oncology. 2016;3(2):e1053594. Epub 2016/06/17. PubMed Central PMCID: PMCPmc4905370. 27308587 10.1080/23723556.2015.1053594 PMC4905370

[12] Kim G, Ison G, McKee AE, Zhang H, Tang S, Gwise T, et al FDA Approval Summary: Olaparib Monotherapy in Patients with Deleterious Germline BRCA-Mutated Advanced Ovarian Cancer Treated with Three or More Lines of Chemotherapy. Clinical cancer research: an official journal of the American Association for Cancer Research. 2015;21(19):4257–61. Epub 2015/07/19. 26187614 10.1158/1078-0432.CCR-15-0887

[13] McCrudden CM, O'Rourke MG, Cherry KE, Yuen HF, O'Rourke D, Babur M, et al Vasoactivity of rucaparib, a PARP-1 inhibitor, is a complex process that involves myosin light chain kinase, P2 receptors, and PARP itself. PLoS One. 2015;10(2):e0118187 Epub 2015/02/18. PubMed Central PMCID: PMCPmc4331495. 10.1371/journal.pone.0118187 25689628 PMC4331495

[14] Lawlor D, Martin P, Busschots S, Thery J, O'Leary JJ, Hennessy BT, et al PARP Inhibitors as P-glyoprotein Substrates. Journal of pharmaceutical sciences. 2014;103(6):1913–20. Epub 2014/04/05. 10.1002/jps.23952 24700236

[15] Sliwoski G, Kothiwale S, Meiler J, Lowe EW Jr. Computational methods in drug discovery. Pharmacological reviews. 2014;66(1):334–95. Epub 2014/01/02. PubMed Central PMCID: PMCPmc3880464. 10.1124/pr.112.007336 24381236 PMC3880464

[16] Cozzini P, Kellogg GE, Spyrakis F, Abraham DJ, Costantino G, Emerson A, et al Target flexibility: an emerging consideration in drug discovery and design. J Med Chem. 2008;51(20):6237–55. Epub 2008/09/13. PubMed Central PMCID: PMCPmc2701403. 10.1021/jm800562d 18785728 PMC2701403

[17] Carlson HA, Masukawa KM, Rubins K, Bushman FD, Jorgensen WL, Lins RD, et al Developing a dynamic pharmacophore model for HIV-1 integrase. J Med Chem. 2000;43(11):2100–14. Epub 2000/06/08. PubMed 10841789 10.1021/jm990322h

[18] Meagher KL, Carlson HA. Incorporating protein flexibility in structure-based drug discovery: using HIV-1 protease as a test case. Journal of the American Chemical Society. 2004;126(41):13276–81. Epub 2004/10/14. 10.1021/ja0469378 15479081

[19] Bowman AL, Nikolovska-Coleska Z, Zhong H, Wang S, Carlson HA. Small molecule inhibitors of the MDM2-p53 interaction discovered by ensemble-based receptor models. Journal of the American Chemical Society. 2007;129(42):12809–14. Epub 2007/10/02. 10.1021/ja073687x 17902662

[20] Meli M, Pennati M, Curto M, Daidone MG, Plescia J, Toba S, et al Small-molecule targeting of heat shock protein 90 chaperone function: rational identification of a new anticancer lead. J Med Chem. 2006;49(26):7721–30. Epub 2006/12/22. 10.1021/jm060836y 17181154

[21] Genoni A, Pennati M, Morra G, Zaffaroni N, Colombo G. Ligand selection from the analysis of protein conformational substates: new leads targeting the N-terminal domain of Hsp90. RSC Advances. 2012;2(10):4268–82.

[22] Colombo G, Margosio B, Ragona L, Neves M, Bonifacio S, Annis DS, et al Non-peptidic thrombospondin-1 mimics as fibroblast growth factor-2 inhibitors: an integrated strategy for the development of new antiangiogenic compounds. The Journal of biological chemistry. 2010;285(12):8733–42. Epub 2010/01/09. PubMed Central PMCID: PMCPmc2838296. 10.1074/jbc.M109.085605 20056600 PMC2838296

[23] Hornak V, Abel R, Okur A, Strockbine B, Roitberg A, Simmerling C. Comparison of multiple Amber force fields and development of improved protein backbone parameters. Proteins. 2006;65(3):712–25. Epub 2006/09/19. PubMed Central PMCID: PMCPmc4805110. 10.1002/prot.21123 16981200 PMC4805110

[24] Wang J, Wolf RM, Caldwell JW, Kollman PA, Case DA. Development and testing of a general amber force field. Journal of computational chemistry. 2004;25(9):1157–74. Epub 2004/04/30. 10.1002/jcc.20035 15116359

[25] Jorgensen WL, Chandrasekhar J, Madura JD, Impey RW, Klein ML. Comparison of simple potential functions for simulating liquid water. The Journal of Chemical Physics. 1983;79(2):926–35.

[26] Darden T, York D, Pedersen L. Particle mesh Ewald: An N⋅log(N) method for Ewald sums in large systems. The Journal of Chemical Physics. 1993;98(12):10089–92.

[27] Hess B, Kutzner C, van der Spoel D, Lindahl E. GROMACS 4: Algorithms for Highly Efficient, Load-Balanced, and Scalable Molecular Simulation. Journal of chemical theory and computation. 2008;4(3):435–47. Epub 2008/03/01. 10.1021/ct700301q 26620784

[28] Lipinski CA, Lombardo F, Dominy BW, Feeney PJ. Experimental and computational approaches to estimate solubility and permeability in drug discovery and development settings. Advanced drug delivery reviews. 2001;46(1–3):3–26. Epub 2001/03/22. PubMed 11259830 10.1016/s0169-409x(00)00129-0

[29] Veber DF, Johnson SR, Cheng HY, Smith BR, Ward KW, Kopple KD. Molecular properties that influence the oral bioavailability of drug candidates. J Med Chem. 2002;45(12):2615–23. Epub 2002/05/31. PubMed 12036371 10.1021/jm020017n

[30] Jorgensen WL, Maxwell DS, Tirado-Rives J. Development and Testing of the OPLS All-Atom Force Field on Conformational Energetics and Properties of Organic Liquids. Journal of the American Chemical Society. 1996;118(45):11225–36.

[31] Kinoshita T, Nakanishi I, Warizaya M, Iwashita A, Kido Y, Hattori K, et al Inhibitor-induced structural change of the active site of human poly(ADP-ribose) polymerase. FEBS letters. 2004;556(1–3):43–6. Epub 2004/01/07. PubMed 14706823 10.1016/s0014-5793(03)01362-0

[32] Hattori K, Kido Y, Yamamoto H, Ishida J, Kamijo K, Murano K, et al Rational approaches to discovery of orally active and brain-penetrable quinazolinone inhibitors of poly(ADP-ribose)polymerase. J Med Chem. 2004;47(17):4151–4. Epub 2004/08/06. 10.1021/jm0499256 15293985

[33] Lee YR, Yu DS, Liang YC, Huang KF, Chou SJ, Chen TC, et al New approaches of PARP-1 inhibitors in human lung cancer cells and cancer stem-like cells by some selected anthraquinone-derived small molecules. PLoS One. 2013;8(2):e56284 Epub 2013/03/02. PubMed Central PMCID: PMCPmc3581553. 10.1371/journal.pone.0056284 23451039 PMC3581553

