# Supplementary figures and images for: Novel PARP-1 Inhibitor Scaffolds Disclosed by a Dynamic Structure-Based Pharmacophore Approach
Source: PLoS One. 2017 Jan 25;12(1):e0170846. doi: 10.1371/journal.pone.0170846 (PMC5266331; doi:10.1371/journal.pone.0170846)

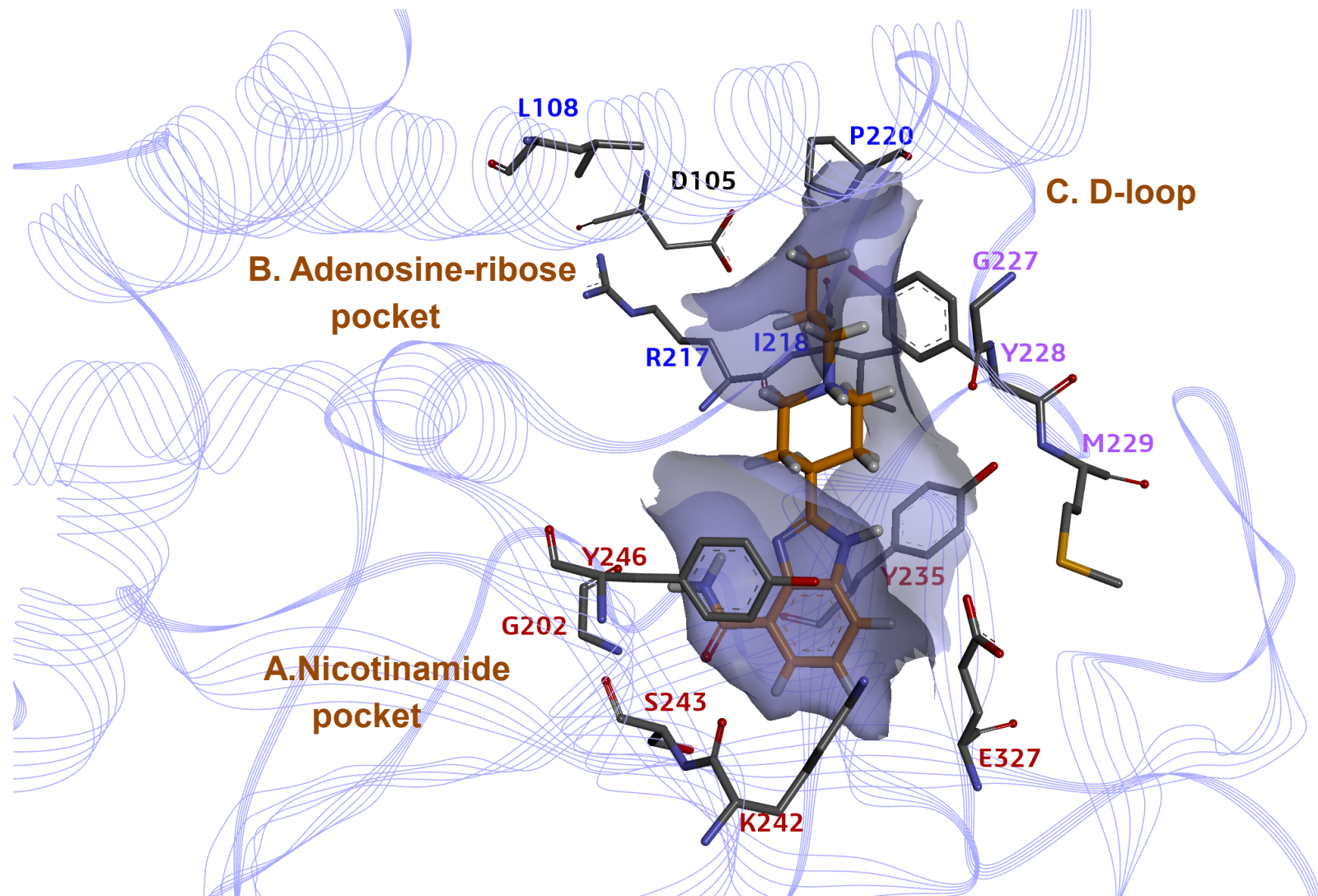

Supplement: S1 Fig — Nicotinamide (A) and adenine-ribose (B) binding residues are displayed in red and blue, respectively. Violet was used to show D-loop residues (C). (PDF) [file pone.0170846.s001.pdf]

3GN7\_crystal

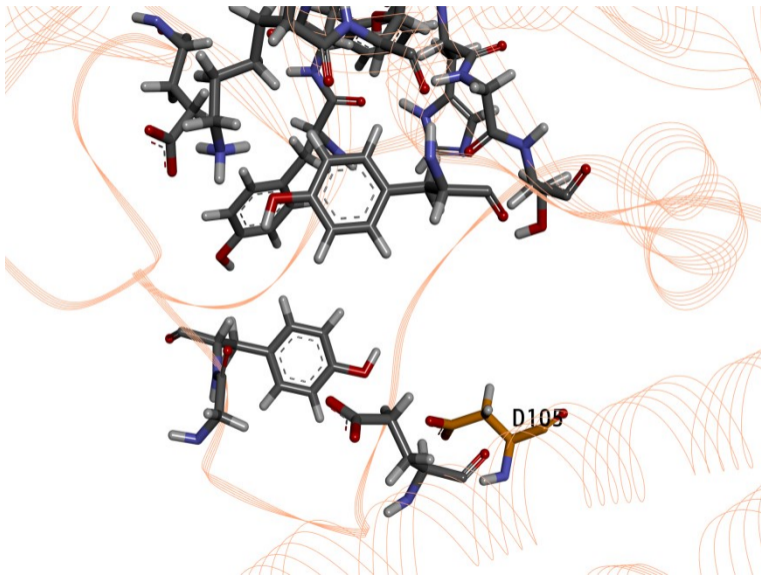

MD\_3GN7

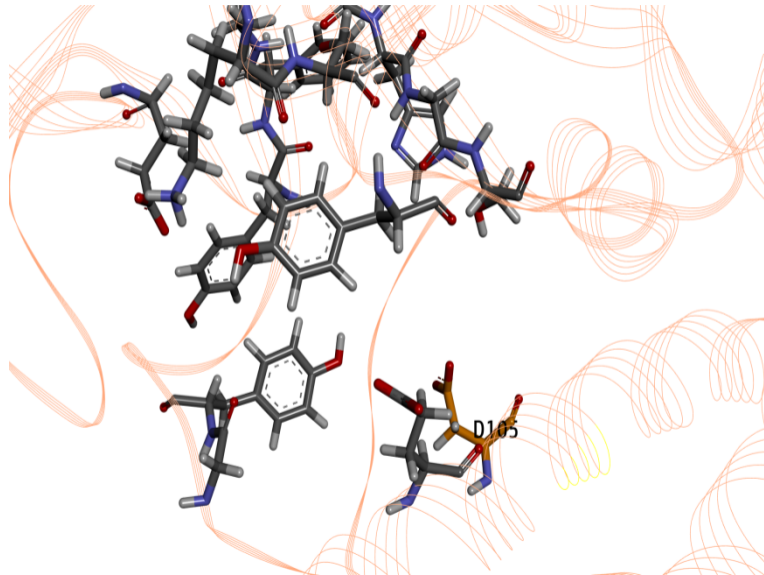

Supplement: S2 Fig — (PDF) [file pone.0170846.s002.pdf]

**S2 File. NOESY spectrum of NSC121848.**

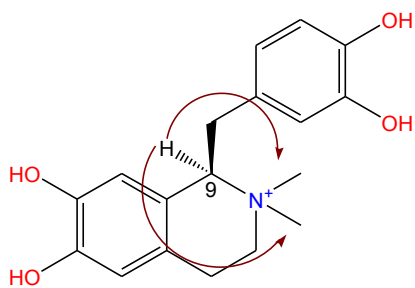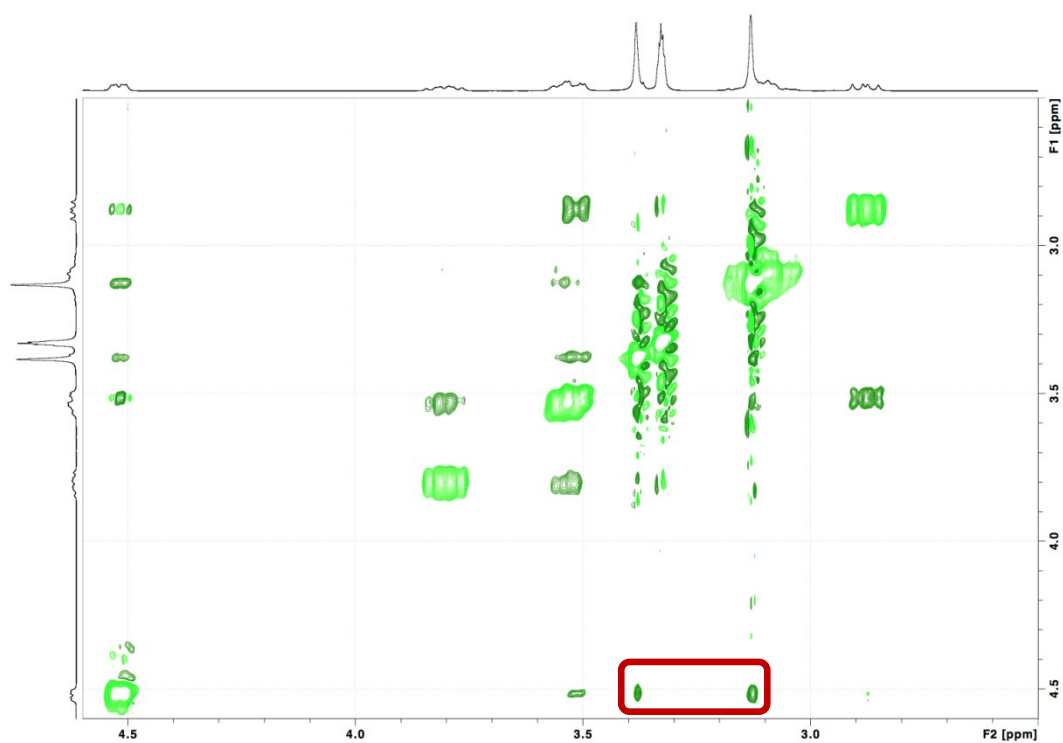

Supplement: S2 File — (PDF) [file pone.0170846.s004.pdf]

**S3 File. NOESY spectrum of NSC86342.**

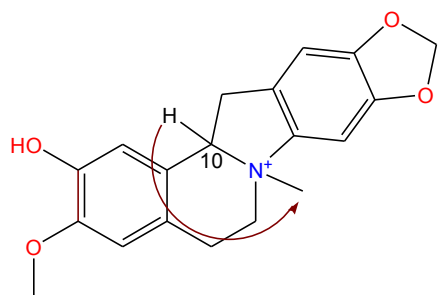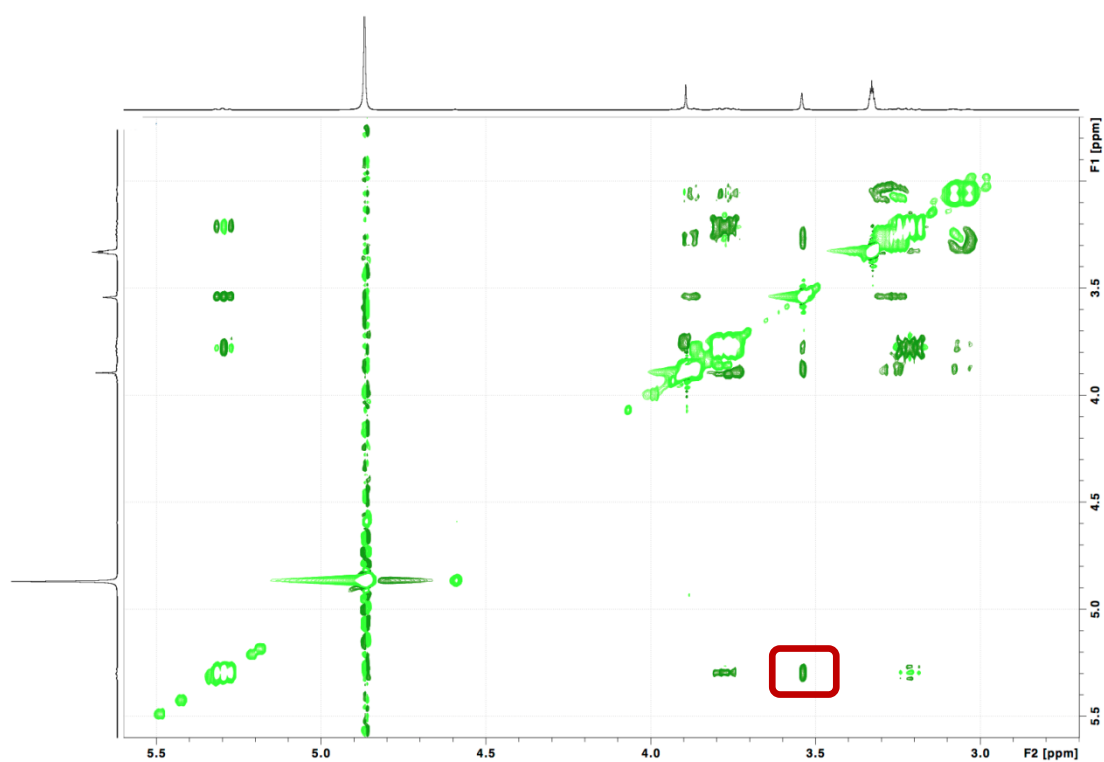

Supplement: S3 File — (PDF) [file pone.0170846.s005.pdf]

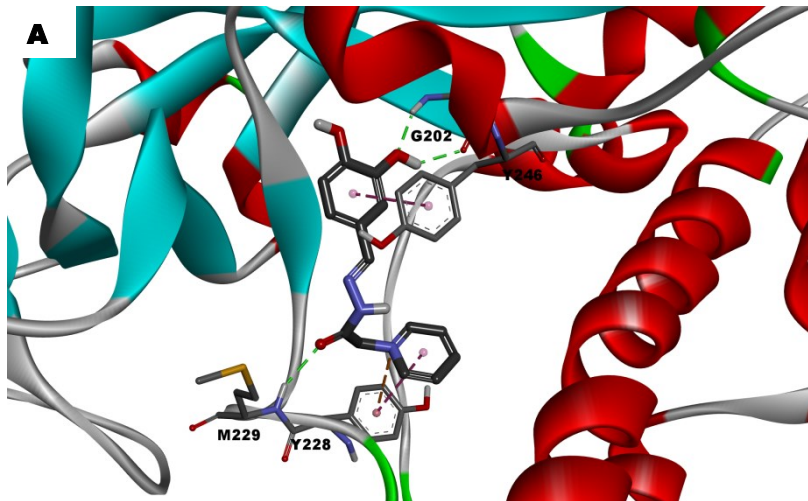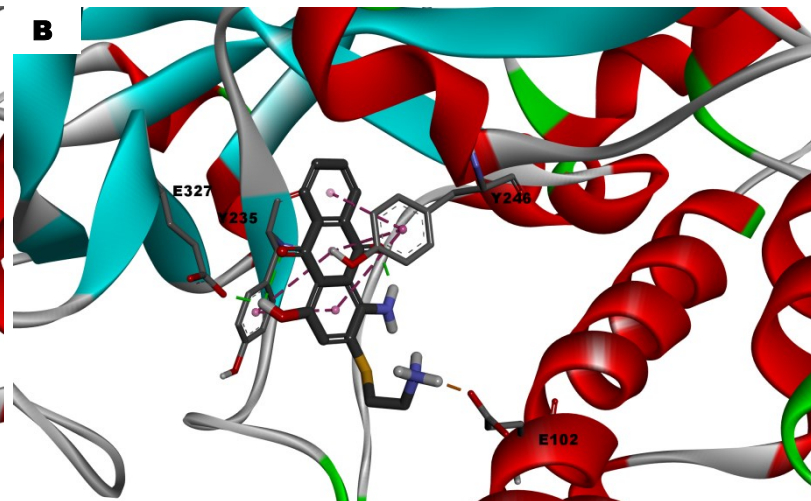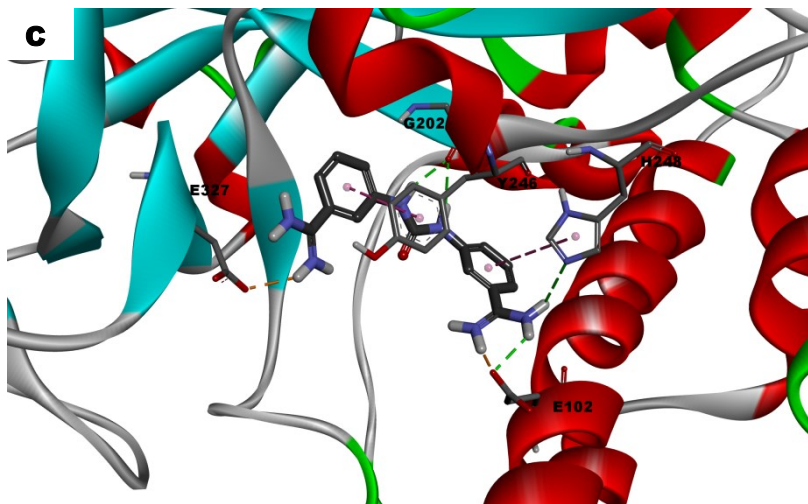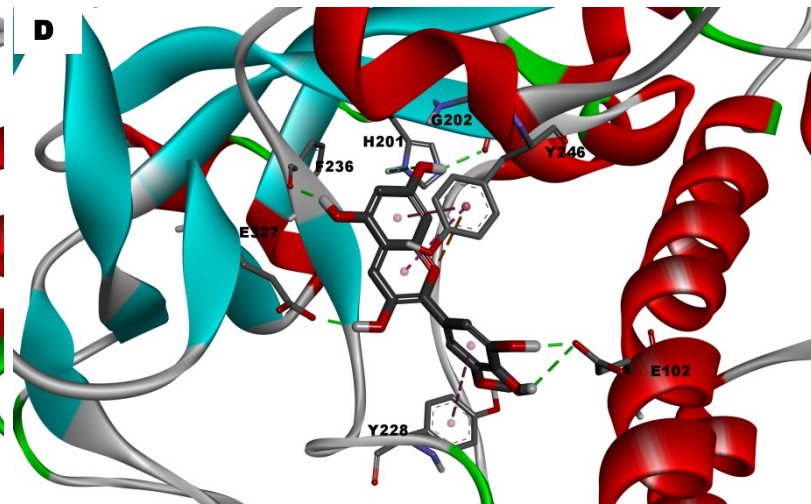

Supplement: S3 Fig — The binding mode of NSC153161 (A), NSC102534 (B), NSC65378 (C) and NSC11907 (D) at the PARP-1 catalytic domain. The molecular interactions of the top scored poses were displayed. (PDF) [file pone.0170846.s006.pdf]
