# Supplementary material for: Novel PARP-1 Inhibitor Scaffolds Disclosed by a Dynamic Structure-Based Pharmacophore Approach
Source: PLoS One. 2017 Jan 25;12(1):e0170846. doi: 10.1371/journal.pone.0170846 (PMC5266331; doi:10.1371/journal.pone.0170846)

S1 File. ^{1}H-NMR and ^{13}C-NMR data for NSC86342, NSC121848 and NSC131753.

1.1 ^{1}H-NMR spectrum for NSC86342 recorded in CD_3OD

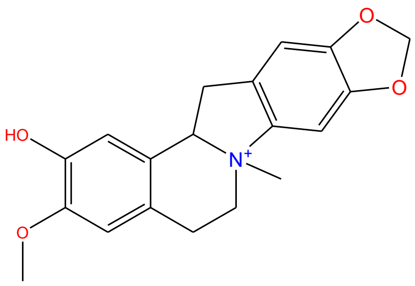

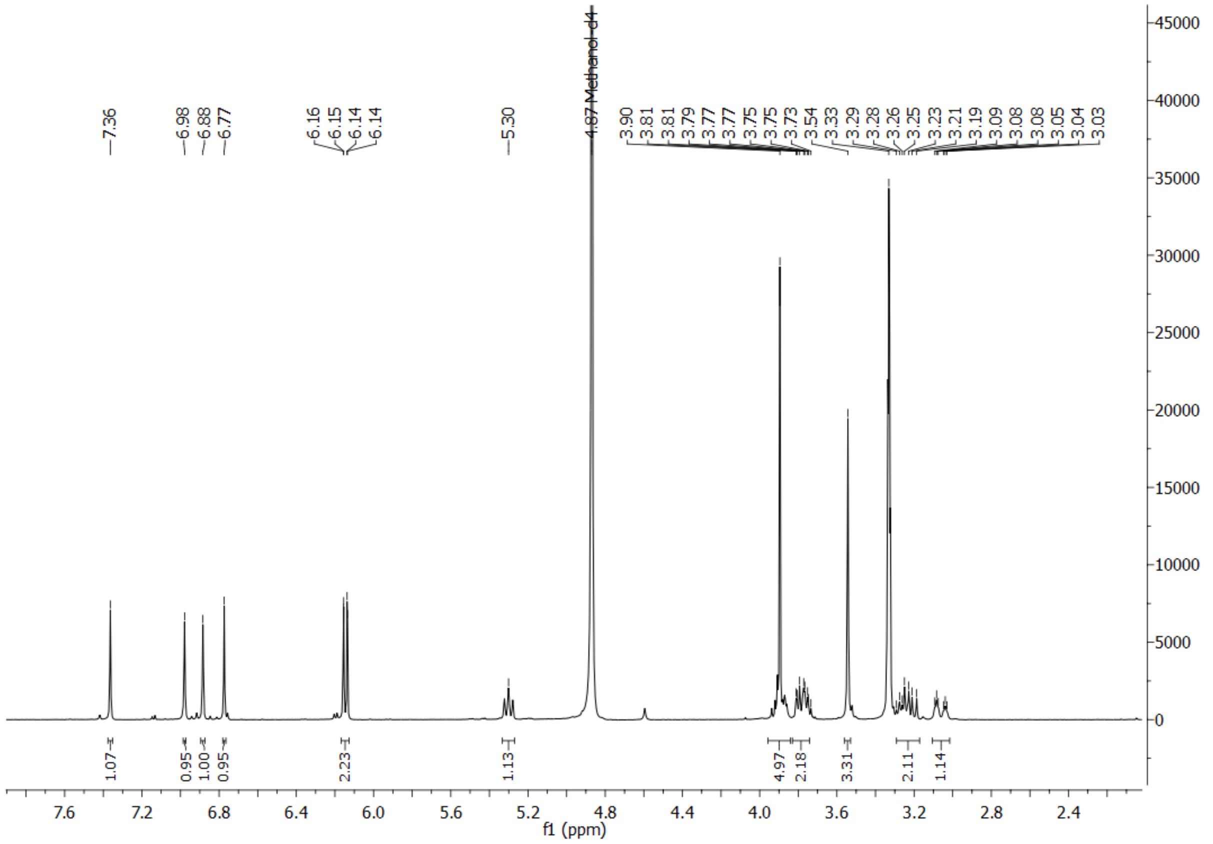

1.2 ^{13}C-NMR spectrum for NSC86342 recorded in CD_3OD

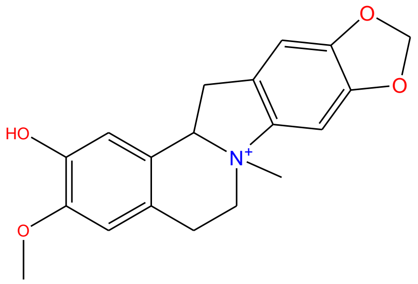

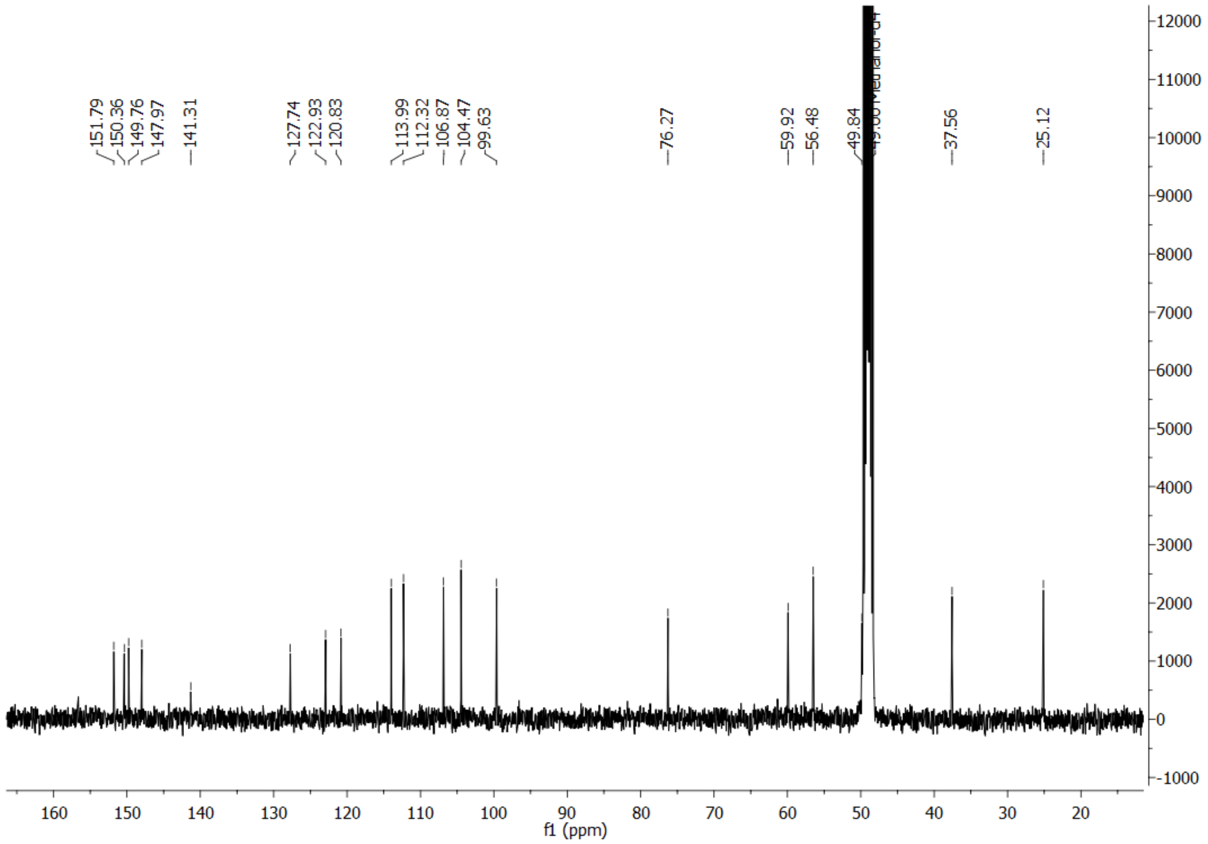

1.3 ^{1}H-NMR spectrum for NSC121848 recorded in CD_3OD

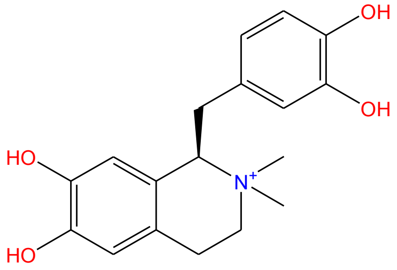

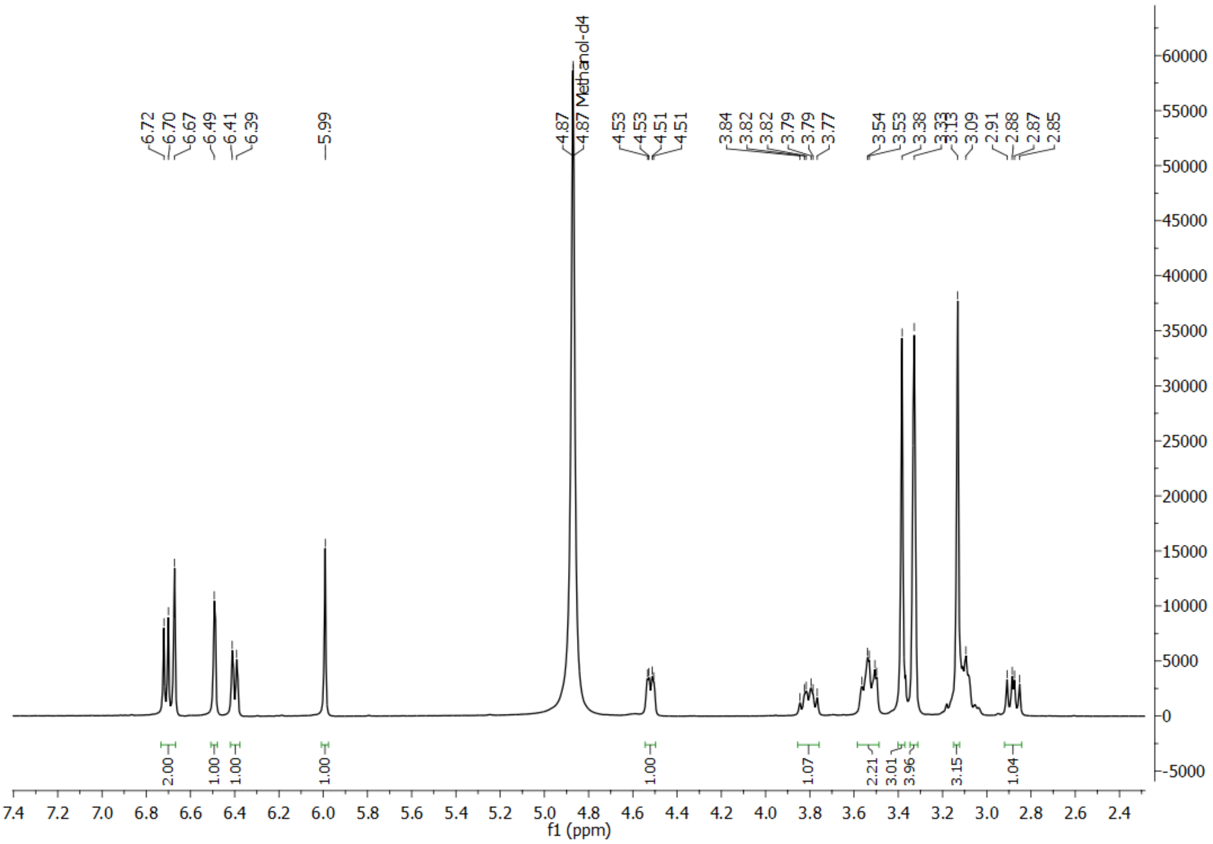

1.4 ^{13}C-NMR spectrum for NSC121848 recorded in CD_3OD

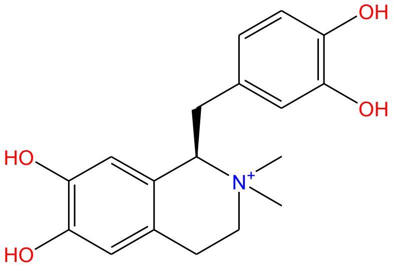

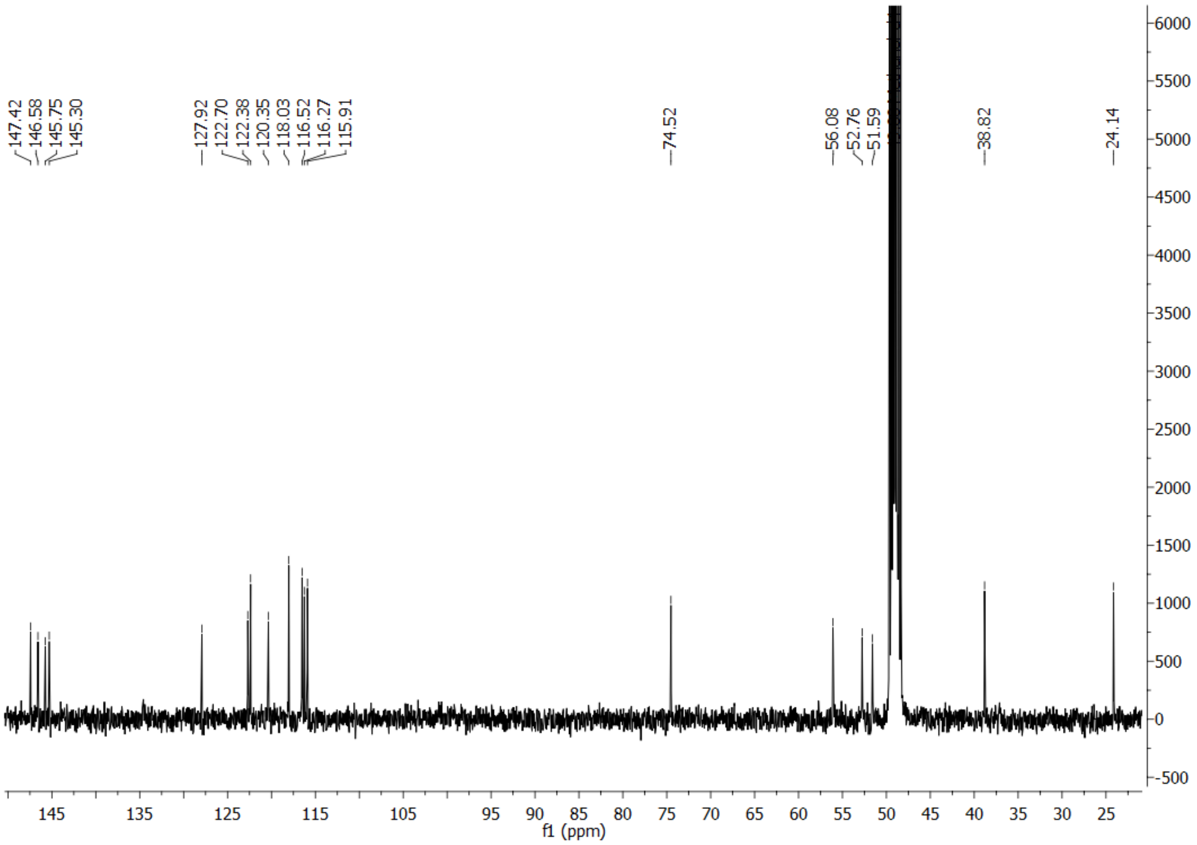

1.5 ^{1}H-NMR spectrum for NSC131753 recorded in CD_3OD

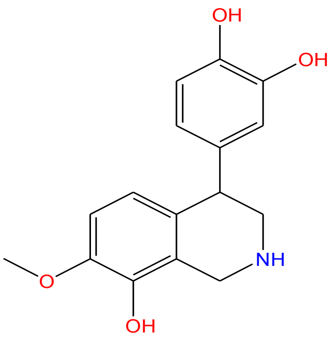

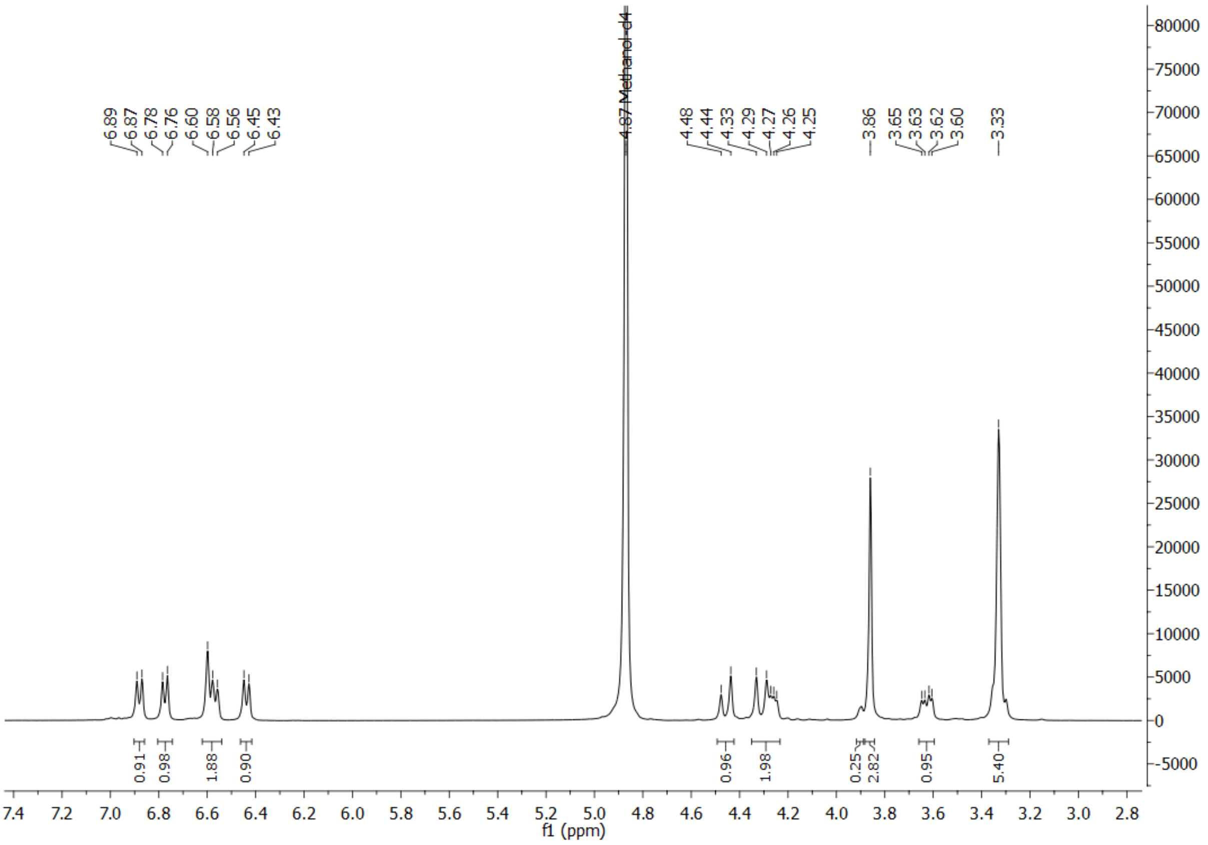

1.6 ^{13}C-NMR spectrum for NSC131753 recorded in CD_3OD

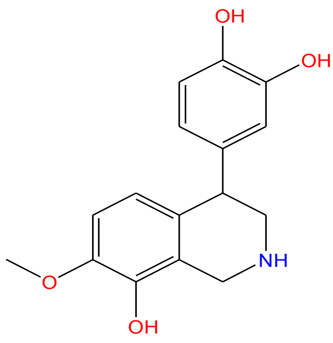

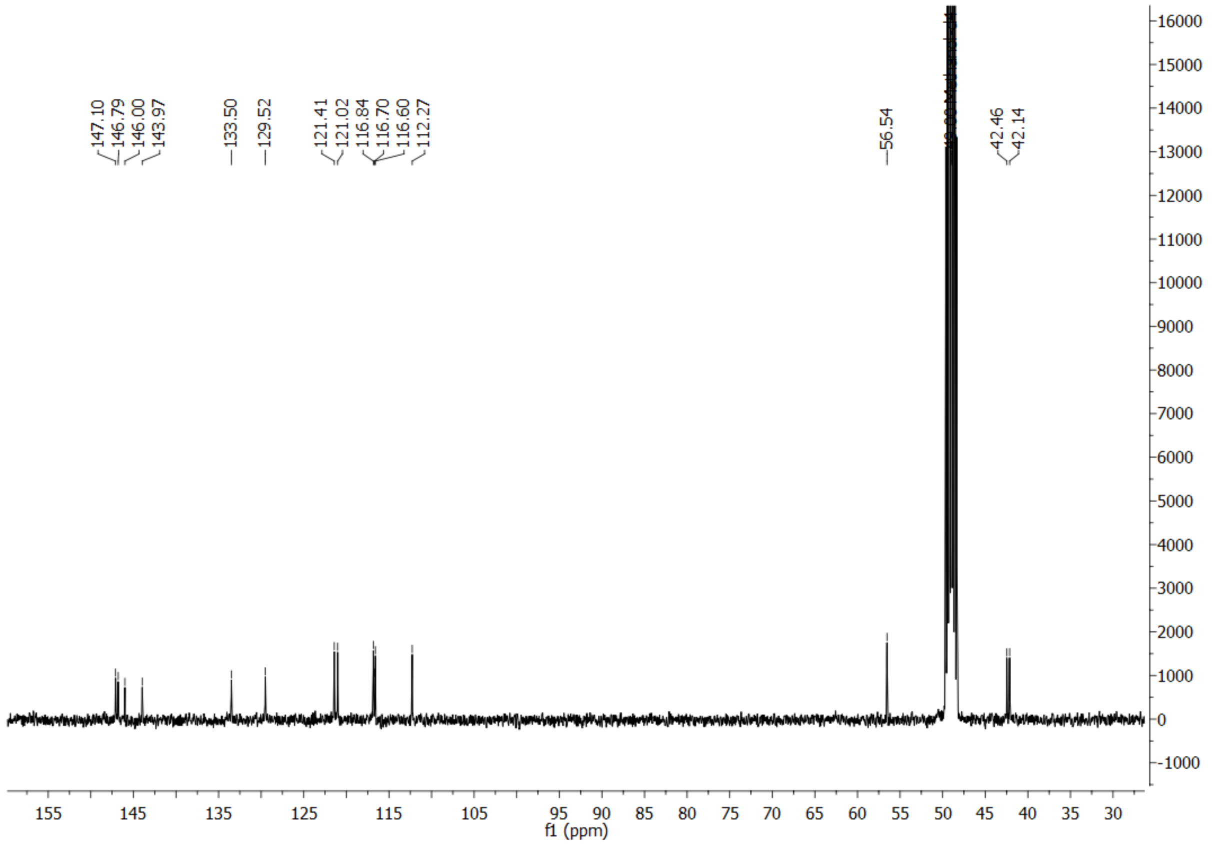

Supplement: S1 File — (PDF) [file pone.0170846.s003.pdf]
